# Assessment of Cancer Therapy Evaluation Program Advocacy and Inclusion Rates of People Living With HIV in Anti–PD1/PDL1 Clinical Trials

**DOI:** 10.1001/jamanetworkopen.2020.27110

**Published:** 2020-12-01

**Authors:** Joshua E. Reuss, Diana Stern, Jared C. Foster, Ramya Ramaswami, Kathryn Lurain, Helen X. Chen, Howard Streicher, Ravie Kem, Richard F. Little, Elad Sharon

**Affiliations:** 1 Lombardi Comprehensive Cancer Center, Georgetown University, Washington, DC; 2 Yale New Haven Health System, Bridgeport, Connecticut; 3 Biometric Research Program, Division of Cancer Treatment and Diagnosis, National Cancer Institute, Bethesda, Maryland; 4 HIV/AIDS Malignancy Branch, National Cancer Institute, Center for Cancer Research, Bethesda, Maryland; 5 Cancer Therapy Evaluation Program, National Cancer Institute, Bethesda, Maryland

## Abstract

**Importance:**

Anti–programmed death 1 and anti–programmed death ligand 1 (anti–PD1/PDL1) immune checkpoint blockade (ICB) constitutes the therapeutic backbone for multiple malignant neoplasms. People living with HIV (PLWH) have routinely been excluded from ICB clinical trials, thus inhibiting broad implementation of ICB to PLWH with cancer.

**Objective:**

To evaluate trends in the inclusion of PLWH in ICB cancer clinical trials that have occurred in association with ongoing efforts by the Cancer Therapy Evaluation Program (CTEP), National Cancer Institute, to promote inclusion of PLWH.

**Design, Setting, and Participants:**

This quality improvement study of ICB letters of intent (LOIs) included anti–PD1/PDL1 agents (nivolumab, pembrolizumab, atezolizumab, and durvalumab) submitted to CTEP that proceeded to approved protocols between January 2014 to May 2019. The setting was ICB clinical trial development and inclusion of underrepresented populations, specifically PLWH. All 97 submitted cancer clinical trial LOIs that included the aforementioned ICB agents were eligible for inclusion. Ten proposals were excluded, of which 3 were designed specifically for PLWH and 7 were LOIs that did not advance to approved protocols within the study period. Statistical analysis was performed from April to September 2020.

**Exposures:**

CTEP advocacy included the requirement for justification of exclusion of PLWH and formal discussion of inclusion criteria during conference calls between CTEP and trial investigators.

**Main Outcomes and Measures:**

The frequency of inclusion of PLWH in initially submitted LOIs was compared with final approved protocols using descriptive statistics. The probability of inclusion of PLWH in submitted LOIs and approved protocols over time was assessed using logistic regression.

**Results:**

Eighty-seven studies were included, of which 68 (78%) were pilot, phase 1, phase 1/2, or phase 2 studies and 19 (22%) were phase 2/3 or phase 3 studies. Thirty-nine studies (45%) included nivolumab, 23 (26%) included pembrolizumab, 19 (22%) included atezolizumab, and 6 (7%) included durvalumab. At initial LOI stage, 14 of 87 (16%) included PLWH. Following CTEP advocacy efforts, 61 of 87 protocols (70%) included PLWH. Of 36 LOIs to initially exclude PLWH, 24 (67%) included PLWH in final protocols. Among the 25 protocols to exclude PLWH, 21 (84%) were earlier phase studies (pilot to phase 2) and 4 (16%) were later phase studies (phase 2/3 to phase 3). Only 13 of 25 protocols (52%) provided justification for exclusion of PLWH, with safety being the most frequently cited concern (9 of 13 studies). The inclusion of PLWH on submitted LOIs increased over time (odds ratio, 3.38; 95% CI, 1.14-3.91), whereas inclusion on final protocols did not increase over time (odds ratio, 1.80; 95% CI, 0.81-1.59).

**Conclusions and Relevance:**

This study identified encouraging trends in the inclusion of PLWH in anti–PD1/PDL1 cancer trials that occurred in the period following the initiation of CTEP advocacy. Work is needed to examine what impact this will have on enrollment of PLWH in such trials. Similar advocacy may help to promote inclusion of other underrepresented populations in cancer clinical trials, including those with organ dysfunction and chronic infections.

## Key Points

**Question:**

Has inclusion of people living with HIV in anti–programmed death 1 and anti–programmed death ligand 1 (anti–PD1/PDL1) immunotherapy trials changed during ongoing Cancer Therapy Evaluation Program advocacy efforts by the National Cancer Institute?

**Findings:**

In this quality improvement analysis of 87 anti–PD1/PDL1 trials approved by the Cancer Therapy Evaluation Program from January 2014 to May 2019, the proportion of studies including people living with HIV increased from 16% of letters of intent to 70% of approved protocols. Inclusion of people living with HIV on submitted letters of intent increased over time.

**Meaning:**

This study’s findings suggest that the increasing inclusion rates of people living with HIV in anti–PD1/PDL1 clinical trials are encouraging and that advocacy for these and other underrepresented populations should continue.

## Teaser

This quality improvement study evaluates trends in the inclusion of people living with HIV in anti–programmed death 1 and anti–programmed death ligand 1 (anti–PD1/PDL1) clinical trials concurrent with ongoing Cancer Therapy Evaluation Program efforts by the National Cancer Institute to promote inclusion of this patient population.

## Introduction

The advent of highly active antiretroviral therapy (ART) has revolutionized the treatment of people living with HIV (PLWH), with many expected to have a nearly normal life expectancy.^1^ Furthermore, the proportion of PLWH in the US aged 65 years or older is projected to increase from 8.5% in 2010 to 21.4% by 2030.^2^ Cancer is now a leading cause of morbidity and mortality in this population,^3^ with non–AIDS-defining cancers, such as lung and colon cancer, becoming increasingly prevalent.^2,4^

In the past decade, the advent of anti–programmed death 1 and anti–programmed death ligand 1 (anti–PD1/PDL1) immune checkpoint blockade (ICB) has transformed the treatment of multiple malignant neoplasms. However, PLWH have routinely been excluded from ICB clinical trials.^5,6^ Although a small number of studies focus exclusively on PLWH, the development of such trials begins a median (range) of 6.3 (3.5-11.7) years after phase 1 trial initiation for a particular therapy.^7^ Recognizing these disparities, in 2017 the American Society of Clinical Oncology (ASCO)–Friends of Cancer Research HIV Working Group published guidelines for modernizing clinical trial eligibility criteria to include otherwise healthy PLWH.^7^ Prior to this, the National Cancer Institute (NCI) Cancer Therapy Evaluation Program (CTEP) began efforts to promote inclusion of PLWH in clinical trials. This culminated in changes to its formal protocol templates in September 2018, at which time clinical and laboratory criteria were recommended to investigators to identify patients with well-controlled HIV who were deemed unlikely to have HIV-related adverse outcomes precluding clinical trial participation.^8^ However, the CTEP advocacy that resulted in this change evolved over time, and the utility of these efforts to promote inclusion of PLWH in ICB cancer clinical trials has not been assessed. We retrospectively reviewed CTEP-approved protocols for anti–PD1/PDL1 agents and compared HIV-specific eligibility criteria in the initially submitted letters of intent (LOIs) with criteria in the final approved protocols to evaluate the evolution in the inclusion of PLWH in anti–PD1/PDL1 ICB clinical trials that occurred concurrently with CTEP advocacy efforts during this time period.

## Methods

This quality improvement study, including manuscript generation, was undertaken in concert with the Standards for Reporting Qualitative Research (SRQR) reporting guideline.^9^ Per guidelines established by the Common Rule, this study was not submitted for institutional review board approval because it did not involve human participants and evaluated clinical trial eligibility criteria prior to any patient enrollment.

LOIs including anti–PD1/PDL1 agents nivolumab, pembrolizumab, atezolizumab, and durvalumab submitted to CTEP that preceded formal CTEP protocol template changes with final study approval between January 2014 and May 2019 were reviewed (eFigure in the Supplement). LOIs approved for protocol development that proceeded to activated clinical trials within the study period were included. Studies designed specifically for PLWH were excluded. The frequency of inclusion of PLWH on initial LOI submission was compared with inclusion on final protocol following CTEP advocacy efforts. Advocacy efforts included requiring justification for exclusion of PLWH and formal discussion of inclusion criteria during conference calls between CTEP reviewers and trial investigators. If trial investigators expressed any reluctance to include PLWH on their protocols, this was brought up during discussion of the reviewed LOIs prior to obtaining CTEP approval to discuss investigator concerns and review the available data on PLWH from other similar trials. These efforts evolved over time. For example, at the start of these advocacy efforts, CTEP initially allowed for a requirement of higher CD4 T cell count thresholds (500 cells/μL) for PLWH compared with other patients in the trial without HIV. Over time, CTEP would suggest lower CD4 cell count thresholds until eligibility requirements were as low as 200 to 250 cells/μL. Ultimately, the requirements for a specific minimum CD4 count threshold were not included in the CTEP protocol template released in September 2018.

### Statistical Analysis

Frequency of PLWH inclusion on submitted LOIs and final protocols was reported using summary statistics. Analyses of the association between calendar time and inclusion of PLWH (both in initial LOIs and approved protocols) were performed. Specifically, explicit inclusion of PLWH (yes vs no or not specified) was modeled as a function of calendar time (in years) using logistic regression. This was done separately for the inclusion of PLWH in initially submitted LOIs and approved protocols. All analyses are descriptive and hypothesis-generating. R statistical software version 3.5.3 (R Project for Statistical Computing) was used for statistical analyses from April to September 2020.

## Results

Eighty-seven approved protocols were included for analysis, of which 68 (78%) were pilot, phase 1, phase 1/2, or phase 2 studies and 19 (22%) were phase 2/3 or phase 3 studies (Table 1 and Figure). Thirty-nine studies (45%) included nivolumab, 23 (26%) included pembrolizumab, 19 (22%) included atezolizumab, and 6 (7%) included durvalumab.

**Table 1.** Characteristics of Included Studies

Characteristic	Studies, No. (%) (N = 87)
Study phase	
Pilot, 1, 1/2, and 2	68 (78)
2/3 and 3	19 (22)
Therapy	
Nivolumab	39 (45)
Pembrolizumab	23 (26)
Atezolizumab	19 (22)
Durvalumab	6 (7)

**Figure.**
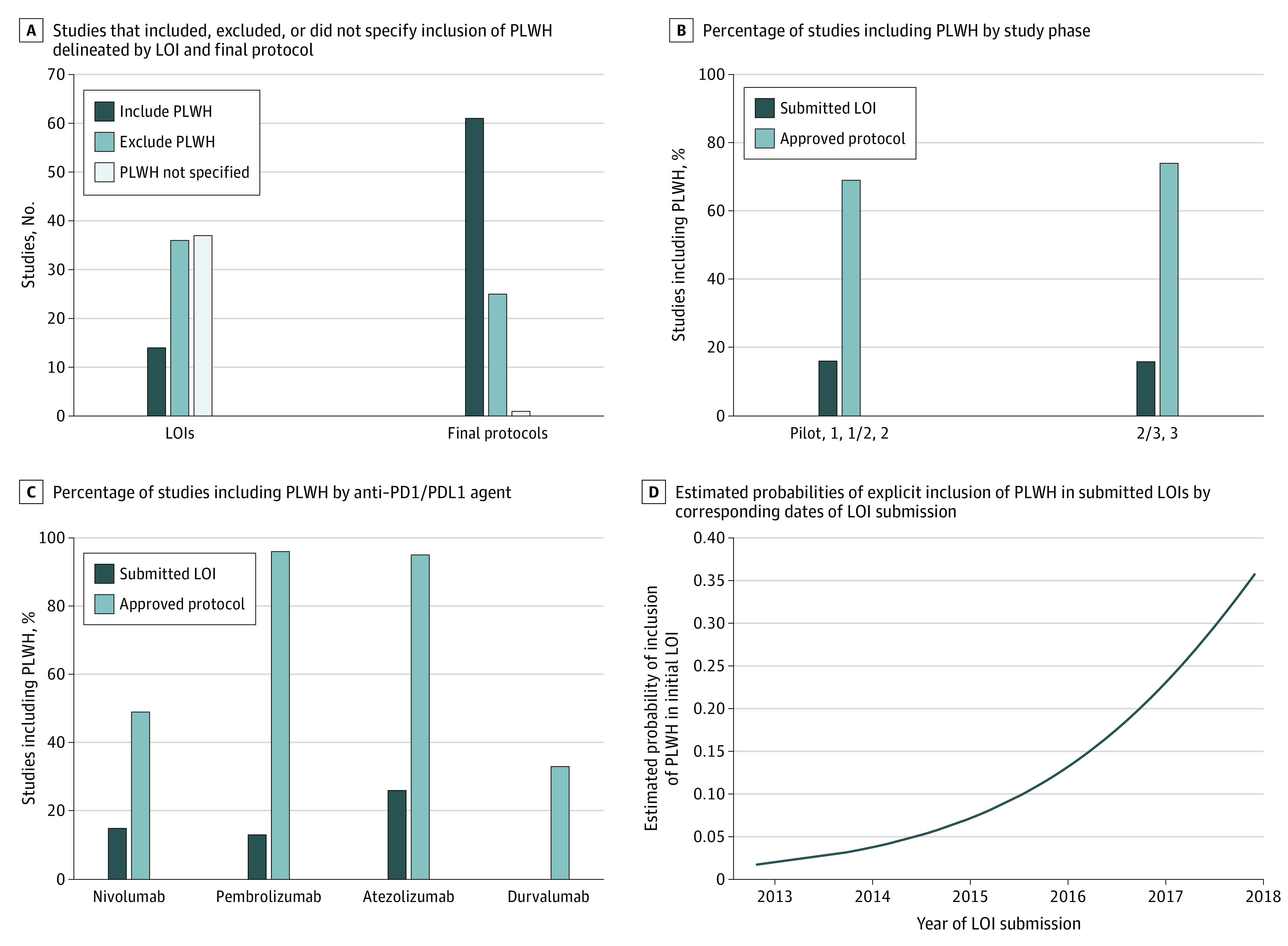
Inclusion of People Living With HIV (PLWH) in Submitted Anti–Programmed Death 1 and Anti–Programmed Death Ligand 1 (Anti–PD1/PDL1) Letter of Intent (LOI) Proposals and Final Protocols Panel A shows raw number of studies that included, excluded, or did not specify inclusion of PLWH delineated by LOI and final protocol. Eighty-seven studies were included. Comparison of inclusion of PLWH in submitted LOI and final protocol is delineated by study phase (B) and anti–PD1/PDL1 agent (C). Panel D shows estimated probabilities of explicit inclusion of PLWH in submitted LOIs (obtained using logistic regression modeling) plotted against corresponding dates of LOI submission.

At the initial LOI stage, 14 of 87 proposals (16%) included PLWH (Table 2 and Figure), including 11 of 68 pilot, phase 1, and phase 2 LOIs (16%) and 3 of 19 phase 2/3 or phase 3 LOIs (16%). Following CTEP policy intervention, 61 of 87 final protocols (70%) included PLWH, including 47 of 68 pilot, phase 1, and phase 2 protocols (69%) and 14 of 19 phase 2/3 or phase 3 protocols (74%). Also, of 36 LOIs that initially excluded PLWH, 24 (67%) included PLWH in their final protocols (data not shown). Consistent increases in inclusion of PLWH were seen across studies of different anti-PD(L)1 agents (Table 2 and Figure). Of the 61 studies to include PLWH, 60 contained HIV-specific eligibility criteria pertaining to baseline allowable CD4 cell count, minimal or undetectable HIV viral load, and/or clinical stability on ART. Thirty-six of 61 trials (59%) contained criteria incorporating all 3 variables with minimum allowable CD4 cell counts ranging from 200 to 500 cells/μL, maximum HIV viral load ranging from undetectable to 14 500 copies/μL, and ART restrictions ranging from none to minimizing overlapping toxicity with study drugs. Twenty-seven studies (44%) excluded PLWH taking antibiotic prophylaxis, and 11 (18%) contained stipulations related to active opportunistic infections or history of AIDS-defining conditions (excluding malignant neoplasm and CD4 count). Of these 11 studies, 4 (36%) excluded PLWH with any history of an AIDS-defining condition (except malignant neoplasm and CD4 cell count). One study mandated a CD4 cell count of greater than 200 cells/μL off all ART without evidence of opportunistic infection for 60 days prior to randomization. Another study required that enrolled PLWH be under the care of an infectious disease specialist, otherwise a formal consult would be made.

**Table 2.** Inclusion of PLWH in Initially Submitted LOIs and Final Approved Protocols

Study and therapy	Studies including PLWH, No. (%)	
	In submitted LOIs	In final protocols
All studies (N = 87)	14 (16)	61 (70)
Study phase		
Pilot, 1, 1/2, and 2 (n = 68)	11 (16)	47 (69)
2/3 and 3 (n = 19)	3 (16)	14 (74)
Therapy		
Nivolumab (n = 39)	6 (15)	19 (49)
Pembrolizumab (n = 23)	3 (13)	22 (96)
Atezolizumab (n = 19)	5 (26)	18 (95)
Durvalumab (n = 6)	0 (0)	2 (33)

Abbreviations: LOI, letter of intent; PLWH, people living with HIV.

Among the 25 protocols to exclude PLWH, 21 (84%) were earlier phase studies (pilot to phase 2) and 4 (16%) were later phase studies (phase 2/3 to phase 3). Only 13 of 25 protocols (52%) provided justification for exclusion of PLWH. Safety was the most commonly cited concern (9 of 13 studies), followed by efficacy in an immunocompromised individual (4 of 13 studies), and study drug-ART interaction (3 of 13 studies). One trial that excluded PLWH noted that HIV testing was not explicitly a criterion for inclusion in the study.

Logistic regression was used to assess the association between inclusion of PLWH in anti–PD1/PDL1 cancer clinical trials and calendar time. Results of these analyses suggested an association between calendar time and LOI inclusion of PLWH (odds ratio, 3.38; 95% CI, 1.14-3.91) but not protocol inclusion of PLWH (odds ratio, 1.80; 95% CI, 0.81-1.59). The association between LOI inclusion of PLWH and calendar time is visually depicted in panel D of the Figure, in which the estimated probabilities of LOI inclusion of PLWH obtained via logistic regression modeling are plotted against corresponding calendar time in years.

## Discussion

We evaluated the evolution of inclusion of PLWH in anti–PD1/PDL1 cancer clinical trials that occurred concurrent with CTEP advocacy efforts. Overall, inclusion of PLWH increased from 16% at the LOI stage to 70% at the final approved protocol stage of development following CTEP advocacy efforts. Increases were observed irrespective of anti–PD1/PDL1 agent or study phase, and inclusion of PLWH on initially submitted LOIs appeared to increase over time.

Despite growing data demonstrating the safety of anti–PD1/PDL1 therapy in PLWH, such patients have routinely been excluded from ICB clinical trials. In our analysis, only 16% of proposals included PLWH at the LOI stage of development. Similarly, in a recent meta-analysis^10^ of published ICB trials including anti–PD1/PDL1 and anti–CTLA-4 (cytotoxic T-lymphocyte–associated protein 4) agents, only 5 of 107 evaluable trials (4.7%) allowed enrollment of PLWH. Potential cited concerns for ICB in PLWH have included the risk of unmasking opportunistic infections and immune reconstitution inflammatory syndrome, as well as the unknown effects of immune stimulatory agents and unclear efficacy in the setting of HIV-associated alterations in the T-cell repertoire.^11,12^ Similar concerns were observed in our analysis, in which protocols that excluded PLWH commonly cited safety, efficacy, and drug-drug interactions as rationale for exclusion of PLWH. This finding is not surprising, as the majority of trials to exclude PLWH in our study (84%) were earlier phase trials (pilot to phase 2), which primarily focus on safety, pharmacokinetics, pharmacodynamics, and early efficacy signals.

Evidence continues to emerge refuting these concerns. Retrospective analyses^13,14^ have demonstrated the safety of ICB in PLWH without new safety signals including increases in opportunistic infections or immune reconstitution inflammatory syndrome. Two recently published prospective studies of the feasibility and safety of PD-1 blockade in PLWH with advanced cancer (DURVAST^15^ and CITN-12^16^) have also reported no new safety signals and demonstrated an ICB toxicity profile similar to that observed in patients without HIV. Critically, perturbations in CD4^+^ and CD8^+^ T-cell counts, as well as HIV viral load, were not observed with ICB treatment. Moreover, encouraging efficacy was seen in both trials, with a clinical benefit rate (defined as tumor shrinkage or stabilization at ≤24 weeks) of 17% with pembrolizumab in the CITN-12 study^16^ and response rate of 25% with durvalumab in the DURVAST study.^15^ Although prospective data powered for clinical efficacy are lacking, these findings suggest ICB is safe in PLWH and also likely has efficacy similar to that observed in biomarker-unselected patients without HIV.

Although 70% of approved protocols ultimately allowed for enrollment of PLWH in our analysis, it is worth noting that all but 1 of these studies (60 of 61) had additional selection criteria most commonly pertaining to a minimum allowable CD4 cell count, undetectable or minimal HIV viral load, and/or clinical stability on ART. The majority of these 61 studies incorporated all 3 variables, but there was significant heterogeneity. Minimum allowable CD4 cell count thresholds ranged from not being part of criteria to 500 cells/μL. In addition, 44% of studies that incorporated PLWH excluded PLWH taking infection prophylaxis and 18% excluded PLWH with any history of an AIDS-defining condition. Not only does this sow confusion over acceptable criteria, but it also creates additional barriers to inclusion of PLWH, limiting potential enrollment. To standardize criteria, in September 2018 CTEP published guidance on inclusion of PLWH, stating that eligibility criteria should be straightforward and focus on current or past CD4 and T cell counts, history of AIDS-defining conditions, and status of HIV treatment.^8^ In addition, formal protocol template language for inclusion of PLWH was recommended, advising investigators to state that “HIV-infected patients on effective anti-retroviral therapy with undetectable viral load within 6 months are eligible”^8^ (eTable in the Supplement). Further research is needed to see whether these guidelines will result in improved uniformity of selection criteria for PLWH in ICB cancer clinical trials.

### Limitations

This study had limitations. One potential limitation of our study is that 37 submitted LOIs did not specify inclusion or exclusion of PLWH; thus, the number of initial LOIs that considered including PLWH may be underreported. Furthermore, although the observed trends in inclusion of PLWH, including an association between calendar time and inclusion of PLWH on anti–PD1/PDL1 LOIs, are promising, it is difficult to determine the extent to which these findings are attributable to CTEP advocacy efforts. Multiple factors likely contributed including advocacy by other groups such as ASCO–Friends of Cancer Research, as well as improved understanding of ICB agents and recognition of their ability to stimulate immune responses in patients with chronic viral infections. Also, although we observed improvements in the integration of PLWH into inclusion criteria for ICB trials, this does not reflect the number of PLWH who were ultimately enrolled in such studies. PLWH are less likely to receive cancer treatment,^17^ and to demonstrate the full impact of our and other advocacy efforts, future studies should examine the number of PLWH referred for participation in ICB trials. Multiple variables likely affect this, including access to health care, use of cancer screening among PLWH and stage of diagnosis, as well as referral practices of primary care practitioners and other subspecialties.

## Conclusions

This quality improvement study identified encouraging trends in inclusion of PLWH in anti–PD1/PDL1 cancer trials that occurred concurrent with CTEP advocacy. These findings suggest that advocacy efforts by CTEP and others may help to meaningfully broaden inclusion criteria for other patient populations underrepresented in cancer clinical trials (eg, organ dysfunction and chronic viral infections).

## Notes

Supplement. **eFigure.** Consort Diagram of Included Studies **eTable.** Cancer Therapy Evaluation Program (CTEP) Guidance on Inclusion of PLWH in Cancer Therapy Protocols
