# Supplementary material for: Assessment of Cancer Therapy Evaluation Program Advocacy and Inclusion Rates of People Living With HIV in Anti–PD1/PDL1 Clinical Trials
Source: JAMA Netw Open. 2020 Dec 1;3(12):e2027110. doi: 10.1001/jamanetworkopen.2020.27110 (PMC7709086; doi:10.1001/jamanetworkopen.2020.27110)
Supplement: Supplement. — eFigure. Consort Diagram of Included Studies eTable. Cancer Therapy Evaluation Program (CTEP) Guidance on Inclusion of PLWH in Cancer Therapy Protocols [file jamanetwopen-e2027110-s001.pdf]

Supplemental Online Content

Reuss JE, Stern D, Foster JC, et al. Assessment of Cancer Therapy Evaluation Program advocacy and inclusion rates of people living with HIV in anti–PD1/PDL1 clinical trials. *JAMA Netw Open*. 2020;3(12):e2027110. doi:10.1001/jamanetworkopen.2020.27110

eFigure. Consort Diagram of Included Studies

eTable. Cancer Therapy Evaluation Program (CTEP) Guidance on Inclusion of PLWH in Cancer Therapy Protocols

This supplemental material has been provided by the authors to give readers additional information about their work.

© 2020 Reuss JE et al. *JAMA Network Open.*

eFigure: Consort Diagram of Included Studies

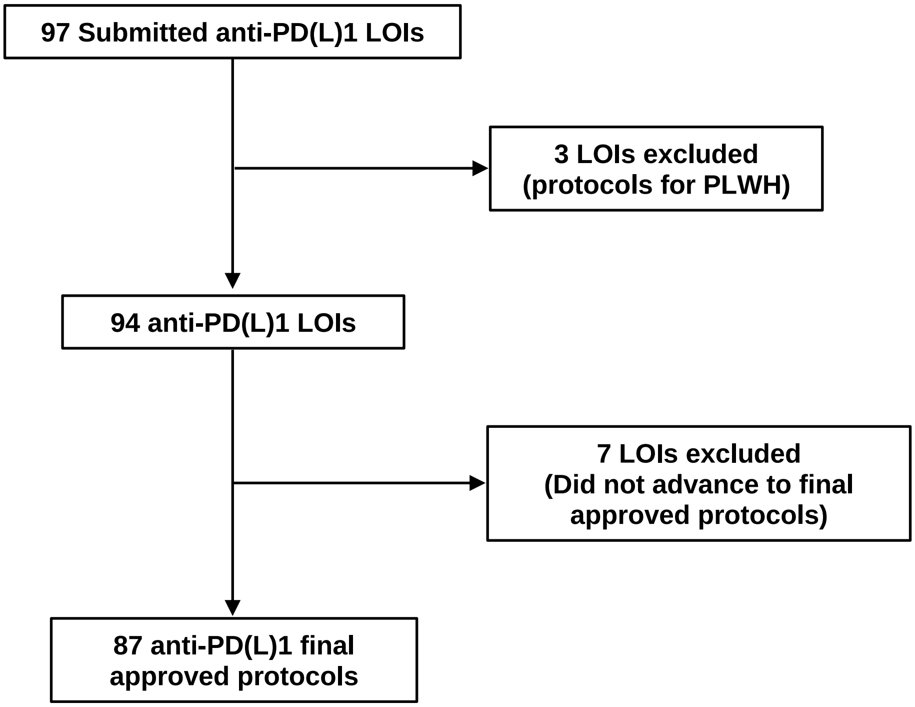

eFigure 1: Consort diagram of included studies. 97 letters of intent (LOIs) including anti-programmed death (ligand) 1 (PD(L)1) therapies were submitted to the National Cancer Institute (NCI) Cancer Therapy Evaluation Program (CTEP) during the study interval. Ten studies were excluded from analysis (3 were designed for people living with HIV (PLWH), 7 did not advance to final approved protocols).

© 2020 Reuss JE et al. *JAMA Network Open.*

eTable 1: Cancer Therapy Evaluation Program (CTEP) Guidance* on Inclusion of PLWH in Cancer Therapy Protocols

CTEP Guidance	ASCO Inclusion Criteria Template Language	CTEP Inclusion Criteria Template Language
HIV-related criteria should be straightforward with focus on:<br>- Current/past CD4 & T-cell counts<br>- History of AIDS-defining conditions<br>- Status of HIV treatment<br><br>Patients with HIV should be treated using identical standards as for other patients with co-morbidities. ART should be considered a concomitant medication.	HIV-infected patients who are healthy and heave a low risk of AIDS-related outcomes are included in this trial.	HIV-infected patients on effective anti-retroviral therapy with undetectable viral load within 6 months are eligible for this trial.

*Adapted from Inclusion/Exclusion Criteria for NCI sponsored clinical trials v.9.26.2018.
ART, Anti-retroviral therapy; ASCO, American Society of Clinical Oncology; CTEP, Cancer Therapy Evaluation Program; PLWH, People living with HIV.

© 2020 Reuss JE et al. *JAMA Network Open.*
